# Association between natural hazards and postnatal care among the neonates in India: a step towards full coverage using geospatial approach

**DOI:** 10.1186/s12873-023-00844-4

**Published:** 2023-07-17

**Authors:** Papai Barman, Nawaj Sarif, Amiya Saha

**Affiliations:** 1 Department of Family & Generations, International Institute for Population Sciences, Mumbai, 400088 India; 2 Department of Migration and Urban Studies, International Institute for Population Sciences, 400088 Mumbai, India

**Keywords:** Natural hazard, Postnatal care, Healthcare accessibility, Spatial analysis, National Family Health Survey

## Abstract

**Background:**

Postnatal care is crucial to prevent the child mortality. Despite the improvement in the PNC coverage for the neonates, it is still far away from the universal health coverage. Along with, some specific regions mostly are natural hazard prone areas of India show very under coverage of PNC for the neonates. Considering the substantial spatial variation of PNC coverage and natural hazard prevalence, present study aimed to examine spatial variation of PNC coverage and its association with natural hazard at the district level.

**Methods:**

The cross-sectional exploratory study utilized National Family Health Survey, 2019-21, which included 1,76,843 children using multistage stratified sampling method to examine postnatal care within 42 days for neonates born within five years prior to the survey. Additionally, the study utilized Vulnerability Atlas of India,2019 maps to categorize regions into hazardous (flood, earthquake, and landslide) and non-hazardous areas. Spatial univariate and bivariate analyses, logistic and geographically weighted regressions were conducted using ArcGIS Pro, GeoDa, and Stata 16.0 software to identify associations between PNC coverage, hazard exposure, and spatial variation.

**Results:**

The univariate spatial analysis showed some specific regions such as north, east, and north-east region of India had a high concentration of natural hazard and low access of PNC coverage. Bivariate analysis also showed that PNC coverage was low in flood (75.9%), earthquake (68.3%), and landslide (80.6%) effected areas. Compared to the national PNC coverage (81.1%), all these natural hazards effected areas showed low coverage. Further, logic regression showed that these hazard prone areas were less (OR:0.85 for flood, 0.77 for earthquake, and 0.77 for landslide) likely to get PNC coverage than their counterparts. LISA cluster maps significantly showed low PNC and high disaster concentration in these disaster-prone areas. Geographic weighted regression results also showed similar result.

**Conclusions:**

The present study elucidates notable heterogeneity in the coverage of postnatal care (PNC) services, with lower concentrations observed in disaster-prone areas. In order to enhance the accessibility and quality of PNC services in these areas, targeted interventions such as the deployment of mobile health services and fortification of health systems are recommended.

## Highlights

• PNC coverage was low in the area affected by flood (75.9%), earthquake (68.3%), and landslide (80.6%).

• All-natural disaster-affected areas had low coverage of Post-Natal Care (PNC) compared to the national PNC coverage (81.1%).

• Focusing at the geographical variation for PNC coverage, all the states and UTs performed well, with the exception of the central, eastern, and northeastern states. Results clearly demonstrated that certain regions—such as the north, east, and northeast regions—performed poorly in terms of PNC coverage because they were more vulnerable.

## Background

Global climate change has led to an increase in both the frequency and severity of natural hazards worldwide. Events such as floods, landslides, storms, heatwaves, and droughts have become more prevalent phenomena [1, 2]. In recent years, Asian countries had a disproportionately large number of natural hazards and disasters compared to other regions of the world [3]. India being an Asian country with a large geographical extension has been experiencing a number of extreme natural hazards and disasters over the past three decades, including floods, landslides, cyclones, earthquakes, droughts, etc., which have devastated a large area of the country [4]. About 170 million of India’s 1.4 billion inhabitants live in the coastal regions, which are particularly vulnerable to the effects of a changing climate due to erosion, sea level rise, and natural disasters like cyclones and tropical storms [5]. Existing landscape of major rivers cause number of floods in different parts of the country, due to excessive rainfall high altitude slope areas experience massive landslide, frequent earthquake in hilly areas etc. The most recent example of this vulnerability happened in May 2020 when Cyclone ‘Amphan’, the deadly storm to hit the Bay of Bengal in decades, flood in Assam and Kerala, landslide in Manipur caused the evacuation of several million people [6]. In fact, as a result of these natural disasters, an increasing number of people are living in poverty, losing their homes and means of support, and becoming landless and homeless [7].

Natural disasters may have various negative effects on public health, including an increase in infectious diseases and injuries, mental stress, a drain on person al and communal resources and infrastructure, and disruptions in health and medical services [8–13]. It has both direct and indirect effects on healthcare systems that limit access to, and availability, price, coverage, and quality of healthcare services [14]. Failure of any component within a system may impact on availability of health care facilities and as a result of several negative things such as maternal, infant, and any other pre-mature mortality may happen [15]. Health check -up by right time always helps to halt such negative things [16].

However, within the health-care check-up system, antenatal (ANC) and postnatal care (PNC) are critical factors corresponding to maternal and child mortality. A Study reported that PNC in disaster prone areas remains a critical problem [17]. PNC refers to special care for the mother and neonate that starts after delivery and lasts for around eight weeks [18]. The care corresponding to neonate includes keeping baby warm, caring for umbilical cord, and checking temperature, cord and danger sign like fever, diarrhoea, nutrition status etc. Inadequate care during this period could lead to illness, impairments, and deaths, and can delay the identification of neonates’ complications and the initiation of appropriate care and treatment [19]. The postpartum period of care helps medical professionals identify post-delivery issues and administer medications appropriately [20]. In India, more than two-thirds of neonatal deaths occur in the first week following birth, and the majority happens during the first 24 h [19]. Therefore, PNC of neonate with an appropriate time is very crucial.

In India, although a majority of states/UTs have improved PNC for neonate over the years 1992-93 to 2021-22, still states/UTs such as Nagaland, Mizoram, Nagaland, Arunachal Pradesh, and Bihar have covered only below 70% in the recent 2021-22 year. According to the recent NFHS-5 report [21], ANMs, nurses, midwives, and LHVs performed the initial postnatal checks for 11% of neonates, compared to 15% of neonates who had a doctor perform the checkup. India has been attempting to inform and educate mothers about maternity and neonate health and coverage of the greatest health check-ups using Mother and Child Protection Card (MCP Card) through the Integrated Child Development Services (ICDS) plan and the National Rural Health Mission (NRHM) [21]. Along with, India by adopting Maternal, neonates, and child health (MNCH) programmes has also advanced towards full coverage of health care utilization over the past ten years, but there is still a significant gap when it comes to addressing MNCH in many areas [22]. The performance in certain regions in India is still low.

The changing healthcare requirements for women who give birth and the neonate in disaster-affected areas have received very little attention internationally [23]. The existing literatures have examined the effects of disaster on maternal health, and healthcare utilization [24–26]. A few studies have examined the postnatal care needs for women who probably end up in stable post-emergency camps, where they are typically given access to food, water, education, and healthcare [27, 28]. Other studies mentioned that infrastructure and medical facilities are frequently damaged during natural hazard and disasters, while the demand for healthcare facilities increases significantly [29–32]. A study from India reported that natural disasters seriously hinder the availability of medical supplies and paediatric healthcare services [33].

However, healthcare accessibility and utilization remain major challenges in the Indian health system. The availability and accessibility of healthcare services vary among different subgroups of the population and across various geographic locations. Across the globe, regions with geographical challenges, such as high-altitude areas, flood-prone areas, and landslide-prone areas, dense forest etc. often experience a lack of access to healthcare facilities. People in these regions face difficulties in accessing health services located far away from their locations. Moreover, preparedness of health system for a hazardous situation is another issue to encounter here. Mostly the preparedness for a long-term impact and resilient health system is very much required based on the vulnerability of a place for an emergency situation [34–36]. Generally, health care utilizations are explained through the socio-economic dimensions of individual but most of previous studies often ignore the challenges in accessing health care due to the natural hazard or challenging environment.

This study introduces location-based analysis of healthcare utilization, perhaps it is a novel approach for studying healthcare related issue using large scale survey data and hazard zonation map. This study will help us to understand that, to what extent natural hazards playing role in healthcare (PNC) utilization in India and point out the requirement for improve health system resilience in geographically challenging locations [37]. India is a multi-hazardous country where a large portion of land gets affected by natural hazard (i.e., flood, landslide, earthquake) frequently [38]. Also, there is very limited study that focused on the spatial heterogeneity in terms of natural hazard and PNC coverage and effect of natural disaster and geographical role on PNC coverage. In India, the trajectory of relationship between these aspects is poorly understood. Therefore, in order to achieve full PNC coverage across the regions, reaching health facility to all and reduce child mortality, it is necessary to understand the prevailing natural hazard in India, affected zones and its effect on the PNC coverage. By addressing the gaps found in the literatures, the current study sought to investigate the spatial heterogeneity of natural hazard and PNC coverage in different regions, and the association between different natural hazard and postnatal care among the babies born in India.

## Materials and methods

### Data source

This cross-sectional exploratory study utilised two secondary data sources and compiled into one dataset; one was National Family Health Survey (NFHS-5) carried out during 2019-21 [21], and other one was The Vulnerability Atlas of India, third edition published in the year 2019 [38]. The NFHS is a nationally representative large-scale survey that has provided information through five consecutive surveys, spanning from 1992 to 1993 to 2019–2021. In the latest round of NFHS 5, approximately 724,115 women aged 15–45 from 636,699 households were covered. The survey achieved a 97% response rate by employing a multi-stage stratified sampling technique. This technique involved two stages for urban areas and three stages for rural areas [21]. It used 2011 census data for the sampling frame to select the Primary Sampling Unit (PSU) in rural and Census Enumeration Blocks (CEBs) in urban areas and at last final PSU and CEB were selected using probability proportional to size (PPS) [21]. It provides reliable information about morbidity, mortality, nutrition, health care and other aspects of mother and child for 707 districts, 28 states, and 8 union territories. Other procedures, including sampling strategy, validation of the data and questionnaire have been comprehensively described in the published report [21]. Whereas, the Vulnerability Atlas of India, 2019 was used to identify the hazardous location in India [38]. The atlas was prepared by the BMTPC (Building Materials, Technology Promotion Council), the ministry of housing and urban affairs. BMTPC atlas is used by Disaster Management authorities, agencies, related stakeholders, and citizens of India to identify the level of the damage risk zone and the degree of vulnerability [38]. This atlas provides maps for multiple hazards such as earthquakes, landslides, floods, wind and cyclones. For preparing this atlas, multiple sources, such as the Indian Meteorological Department (IMD), Survey of India (SOI), Geological Survey of India (GSI), Census of India, Bureau of Indian Standards (BIS), and Central Water Commission (CWC) were used [38].

From the available dataset flood, landslide and earthquake high risk hazard zonation maps were prepared. Flood, landslide, and earthquake were considered frequent and severe threat in most part of the country, therefore in this study we considered these three natural hazards. We first prepared the vector files of the hazard map of earthquake, landslide, and flood, and then the NFHS clusters were used to categorise the PSU and household of any areas into the hazardous and non-hazardous categories. Cluster points situated in flood, landslide, and earthquake high-risk zones were considered as hazardous cluster points while others were non-hazardous. Therefore, the households in that PSU were considered as affected by floods, landslides, and earthquakes.

### Study sample

Based on the study objectives, the sample for the study was restricted based on the following criteria: (1) the child must have been born in the five years preceding the survey, (2) the child must have been alive at the time of the survey, and (3) the child must have been the last child born to the mother. As a result, the final sample size for the study was 176,843 children.

### Variable description

#### Outcome variable

The outcome variable of the current study was post-natal check within 42 days. NFHS-5 included question about whether the mother’s last child received a post-natal check before 42 days after birth and before discharge from the facility where the delivery took place. The survey also asked about the time for post-natal care took place for after delivery and before discharge, and whether the check was conducted by a doctor, auxiliary nurse midwife, or lady health visitor. In this study, a value of 1 was assigned if the child received any post-natal check by a skilled health provider either after delivery or before discharge before 42 days, and a value of 0 was assigned otherwise as per recommendation of the World Health Organization [39, 40]. Since, any natural hazard has a long-time effect on any health care accessibility, we assumed that considering 42 days will be appropriate for better capturing the effect and geographic variations.

### Control variables

Considering most prevailed natural hazard and data limitations in India, we utilized key independent variables such as exposure to flood, earthquake and landslide categorized as “yes” and “no”. Along with, some socio-economic characteristics were also included. It is observed that socio-economically advanced or better-positioned people or family either change their place to hazard risk free areas or use some safety measures to overcome the problems related to any hazard and access to health care, while better-off people or families could not. Keeping the importance of socio-economic disparity, we utilised place of residence (rural and urban) [41], religious category (Hindu, Muslim, Christian and other) [41], social category (SC and ST, OBC and others) [41], educational level (no education, primary, secondary, and higher) [41], wealth quintile (poorest, poorer, middle, richer and richest) [42], mass media exposure (no and yes) [43].

### Statistical approach

Bivariate analysis was carried out to examine the bivariate association and following that, chi-square test was employed to understand the statistical difference among the indicators. To investigate the association between a key independent and the outcome variables after with and without adjusting for socioeconomic condition, logistic regression was also employed. All the analyses were carried out using STATA 16.0 (StataCorp).

### Spatial analysis

Software such as GeoDa version 1.6.7 and ArcGIS version 10.8 was employed for mapping and spatial analysis. Univariate and Bivariate local Moran’s I statistics, LISA significance map and scatter plot were generated for the PNC with other predictor variables such as percentage of household exposed to flood, landslide, and earthquake at the district level using GeoDa software. Moran’s I is a metric of spatial autocorrelation which is a generalization of Pearson’s correlation coefficient. It was used to assess the spatial dependences of PNC coverage in the districts of India. To calculate this spatial autocorrelation, a spatial weight matrix was generated using the queen contiguity method. This matrix generates spatial proximity between each potential set of districts. The following formulas were adopted to calculate the Univariate and Bivariate locals Moran’s I:

$$\mathbf{Univariate}\;\mathbf{Local}\;\mathbf{Moran}\boldsymbol'\mathbf{s}\;\mathbf{ I}\;\boldsymbol{=}\;\frac{\boldsymbol n}{{\boldsymbol S}_{\mathbf0}}\;\boldsymbol{\ast}\;\frac{\boldsymbol{\sum}\boldsymbol {i}\;\boldsymbol{\sum}{\boldsymbol{jw}}_{\boldsymbol{ij}}{({\boldsymbol x}_{\boldsymbol i}\boldsymbol{-}\overline{\mathbf x})}\;{({\boldsymbol x}_{\boldsymbol j}\boldsymbol{-}\overline{\mathbf x})}}{\boldsymbol{\sum}\boldsymbol{i}\;{({\boldsymbol x}_{\boldsymbol i}\boldsymbol{-}\overline{\mathbf x})}^{\mathbf2}}$$

Here, x and is the variables of interest, X̄ refers to the mean of x, n means the number of spatial units, w_ij_ means the standardized weight matrix between observation I and j with zeroes on the diagonal, and S_0_ refers to all aggregated spatial weights, i.e., S_0_=∑_i_∑_j_w_ij_.

$$\mathbf{Bivariate}\;\mathbf{Local}\;\mathbf{Moran}\boldsymbol{'}\mathbf{s}\;\mathbf{I}\;\mathbf{=}\frac{\boldsymbol n}{{\boldsymbol S}_{\boldsymbol{0}}}{\ast}{\;}\frac{\sum \boldsymbol{i}\;\sum \boldsymbol{j}{\boldsymbol{ W}}_{\boldsymbol{ij}}\;{(\boldsymbol{x}_{\boldsymbol{i}}-{\overline{\boldsymbol x}})\;{(\boldsymbol{y}_{\boldsymbol{j}}-\overline{\boldsymbol Y})}}}{\sum \boldsymbol{i}\;{(\boldsymbol{y}_{\boldsymbol{i}}-\overline{\boldsymbol Y})}^{\boldsymbol{2}}}$$

Here, x and y are the variables of interest; X̄ refers to the mean of x, Ȳ refers to the mean of y, n means the number of spatial units, w_ij_ means the standardized weight matrix between observation i and j with zeroes on the diagonal, and S_0_ refers to all aggregated spatial weights, i.e., S_0_=∑_i_∑_j_w_ij_.

#### Hot spots

Districts with high values surrounded by the district with high values (High-High).

#### Cold Spots

Districts with low values surrounded by the district with low values (Low-Low).

#### Spatial outliers

Districts with high values surrounded by the district with low values (High-Low) and districts with low values surrounded by the district with high values (Low-High).

Univariate Local Moran’s I was implemented to understand spatial cluster of high and low access to PNC. While Bivariate LISA analysis was performed to understand the spatial association of PNC coverage gap with other natural hazard indicators. These indicators are percentage of individual exposed to flood, landslide, and earthquake in the district. While the significance map was used to locate the district where the association is significant, and the Moran’s I scatter plot to show the spatial dependence among the variables. Further, Geographically Weighted Regression (GWR), a local form of linear regression was used to model spatially varying relationships among the indicators-PNC, exposure to flood, landslide and severe earthquake for the districts of India.

## Results

**Fig. 1:**
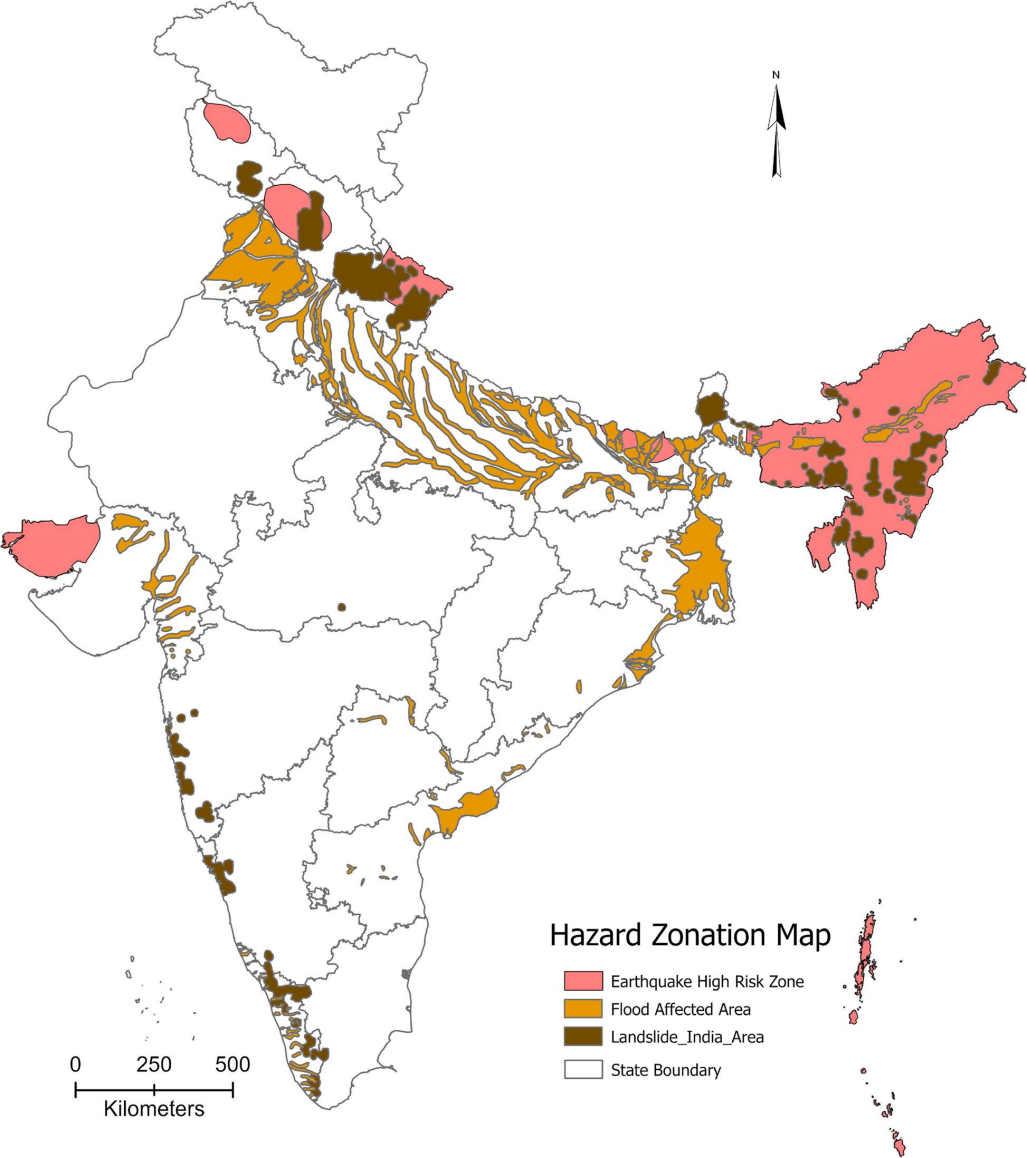
Flood, landslide and earthquake high risk hazard zonation map of India. *Source*: Prepared by the authors’ (compiled using the map from Vulnerability Atlas of India, 2019). Note: https://vai.bmtpc.org/

The combined multiple hazard zonation map portrayed the areas where a number of hazard risk can be found (Fig. 1). It identified the locations of natural hazard that includes flood, landslide, and earthquake (very high damage risk zone). Places can be identified where hazard risk were very minimal while some places were exposed to multiple hazards. Some states were highly affected by these hazards, especially in lower Himalayan belt covering north-eastern, eastern, central and northern part of India. While in the west coastal belt, landslides and floods were very prominent. On the other hand, the area under hazard zones was very low in among major states in the central part country.

The bivariate association between PNC coverage and the different types of natural hazards is presented in Table 1. A significant difference in PNC coverage was found in the flood and earthquake affected areas. Almost 7% difference was observed in flood effected area, whereas it was 24% in earthquake effected area. A minor difference was observed between landslide effected and not-effected area. Further, a substantial gap was observed across the number of hazards, showing lower PNC coverage with increasing number of hazards. For instance, PNC coverage was found to be 81.9% in non-effected areas, whereas it was 65.9% in the area experiencing three all three hazards (Table 1).

**Table 1:** Bivariate association between PNC coverage and natural hazards

Hazards	No coverage	Coverage	n
Flood hazard	Chi = 71.16, *p* < 0.000		
0	17.6	82.4	151,512
1	24.2	75.9	25,331
Earthquake hazard	Chi = 392.26, *p* < 0.000		
0	17.9	82.1	129,774
1	31.7	68.3	47,069
Landslide hazard	Chi = 957.46, *p* < 0.000		
0	18.9	81.1	166,085
1	19.4	80.6	10,758
Number of hazards	Chi = 0.0004, *p* < 0.000		
0	18.1	81.9	110,038
1	19.7	80.3	46,124
2	26.8	73.2	16,853
3	34.1	65.9	3828
Total	18.9	81.1	176,843

*Source:* Author’s computation from NFHS 5 data

Logistic regression estimates show the association between natural hazards and its effect on the PNC coverage (Table 2). The results showed that children in flood effected areas were 0.15 times (OR: 0.85; CI: 0.83, 0.89) less likely to get PNC coverage than non-flood areas compared to the reference category. Similarly, children in the earthquake and landslide effected areas were also 0.23 (OR: 0.77; CI: 0.73, 0.82) and 0.23 times (OR:0.77; CI: 0.73, 0.81) respectively lesser likely to get PNC coverage. It was also found that children in East (OR: 0.62; CI: 0.59, 0.64), North-East (OR: 0.51; CI: 0.48, 0.55), and Central region had (OR: 0.60; CI: 0.58, 0.63) lesser likely to get coverage of PNC in compared to the reference category. The children among the Muslim, Christian and other religious group were less likely to get the coverage of PNC 0.19 (OR: 0.81; CI: 0.78, 0.84), 0.53 (OR: 0.47; CI: 0.45, 0.50) and 0.33 times (OR: 0.67; CI: 0.63, 0.72), respectively. While children among the mothers with higher education were more likely to get PNC coverage (OR: 2.01; CI: 1.91, 2.12). Areas with high media exposure were 0.53 times (OR: 1.53; CI: 1.49, 1.58) more likely to get PNC coverage than non-media exposure.

**Table 2:** Logistic regression estimates the association between natural hazards and PNC coverage

Characteristics	Odds Ratio	CI at 95%	
Hazard			
Flood	0.85*	0.83	0.89
Earthquake	0.77*	0.73	0.82
Landslide	0.77*	0.73	0.81
Geographic region			
North			
East	0.62*	0.59	0.64
Northeast	0.51*	0.48	0.55
Central	0.60*	0.58	0.63
West	1.29*	1.21	1.38
South	1.41*	1.33	1.50
Place of residence			
Rural			
Urban	1.00	0.97	1.04
Religion category			
Hindu			
Muslim	0.81*	0.78	0.84
Christian	0.47*	0.45	0.50
Other	0.67*	0.63	0.72
Social category			
SC and ST			
OBC	1.01	0.98	1.04
Other	0.98	0.95	1.02
Educational level			
No education			
Primary	1.30*	1.25	1.35
Secondary	1.69*	1.63	1.74
Higher secondary	2.01*	1.91	2.12
Wealth quintile			
Poorest			
poorer	1.27*	1.23	1.31
Middle	1.49*	1.43	1.55
Richer	1.68*	1.60	1.76
Richest	2.17*	2.04	2.31
Mass media exposure			
Not exposed			
Exposed	1.53*	1.49	1.58
Constant	2.43*	2.32	2.54

Individual characteristics were considered for mothers

*Source:* Author’s computation from NFHS 5 data

*p < 0.01

**Fig. 2:**
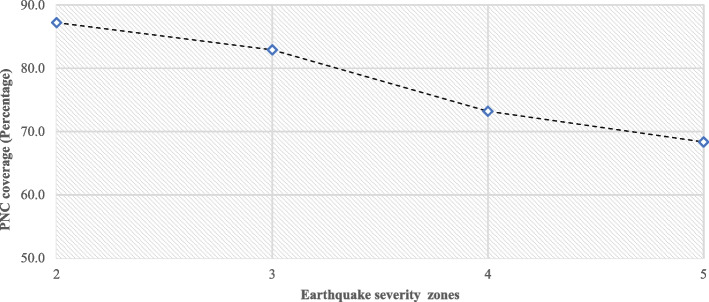
Differences in PNC in different earthquake hazard risk zones in India, 2019-20. *Source*: Author’s computation from NFHS 5 data

Figure 2 depicts the level of PNC (perinatal care) in earthquake hazard risk zones. The level of PNC declines as the risk increases. For example, in Zone 5 (very high-risk zone), the PNC level was 68%, whereas in Zone 2 (low-risk zone), it was 87% (Fig. 1). Univariate and bivariate Moran’s I value was used to determine the spatial association between PNC access and the percentage of individuals exposed to flood, landslide, and severe earthquakes. The Moran’s I value was 0.705 for PNC, 0.069 for flood and PNC, 0.498 for severe earthquakes and PNC, and 0.022 for landslides and PNC. These values indicate positive spatial autocorrelation among the indicators (Table 3).

**Table 3:** Moran’s I value of univariate and bivariate LISA cluster map of PNC and other indicators in India 2019-21

Indicators	Moran’s I Value	*p*-value
PNC	0.705	0.01
Per cent individual exposed to Flood and PNC	0.069	0.01
Per cent individual exposed to severe Earthquake and PNC	0.498	0.01
Per cent individual exposed to Landslide and PNC	0.022	0.01

**Fig. 3:**
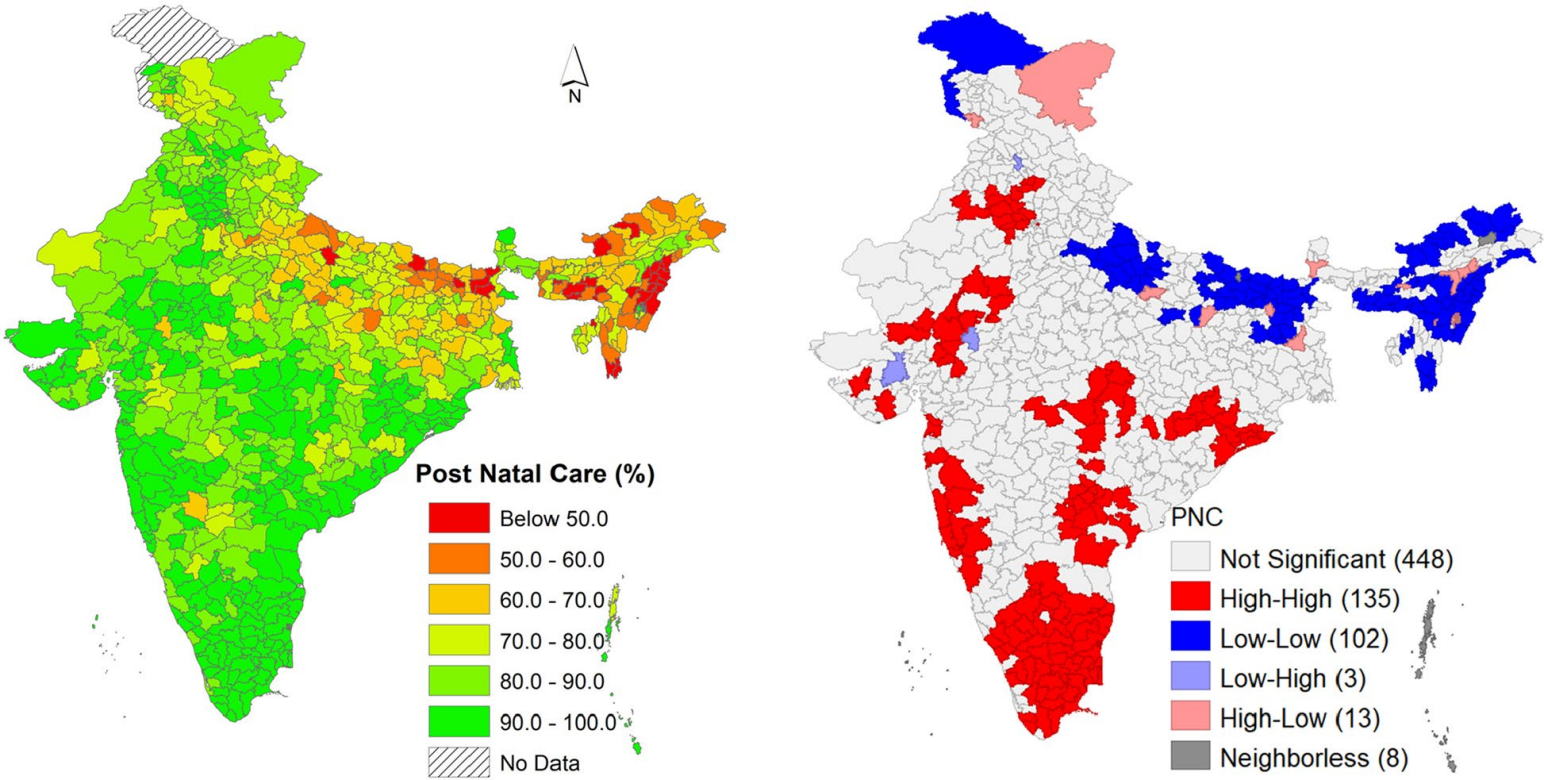
PNC Distribution map and Univariate LISA cluster map of PNC in India, 2019-21. *Source*: Author’s computation from NFHS 5 data

Figure 3 presents the distribution map of PNC for neonates and the LISA cluster map. In the first map, it was observed that central, eastern and north-eastern and central part of India have low access to PNC for neonate compared to other parts of India. The LISA clusters map presented a category of four groups like High-High cluster (Red colour) also can be mentioned as Hot-spot, low-low cluster (Blue colour) known as Cold-spot, and High-low cluster and low-high cluster are the outliers in the map. Out of 707 districts, 135 were coming in the hotspot zones, and 102 were in the cold spot zones. Hotspots can be identified in the selected districts of Haryana, Maharashtra, Kerala, Andhra Pradesh, Odisha and Tamil Nadu where PNC is higher in individual and surrounding districts. Whereas selected districts in states like Uttar Pradesh, Bihar, Arunachal Pradesh, Assam, Nagaland and Manipur shows low-low (102 districts) clusters of Post Natal Cares (PNC) for neonates (Fig. 3).

**Fig. 4:**
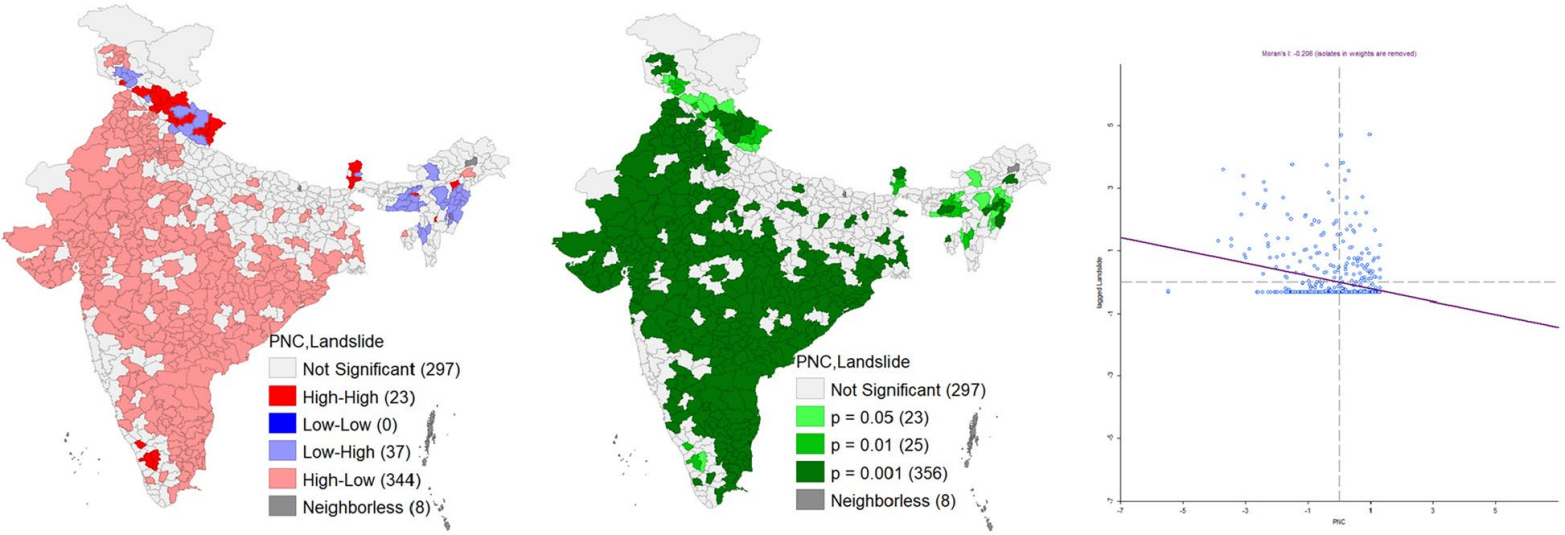
Bivariate Lisa cluster map, significance map and Moran’s-I scatter plot of PNC and Landslide in India. *Source*: Author’s computation from NFHS 5 data

Figure 4 represents the bivariate LISA cluster map; corresponding significance map and the Moran’s-I scatter plot of PNC and Landslide in India. It shows that 23 districts were in the cluster of high-high, and 37 districts in the low-high category. However, 344 districts registered in the higher PNC with low risk of landslide (high-low cluster). High-high district clusters were located in Himachal Pradesh, Sikkim and Uttarakhand, while low-high district clusters are located in Meghalaya, Nagaland, Manipur, Uttarakhand etc. It also shows that 356 districts where spatial autocorrelations can be observed with a significance level of 0.1% (Fig. 4). Additionally, Moran’s Scatter plot indicates strong negative spatial autocorrelation with spatial clusters exists. This infers that a lower level of PNC is clustered where people are exposed to landslide hazard and vice versa.

**Fig. 5:**
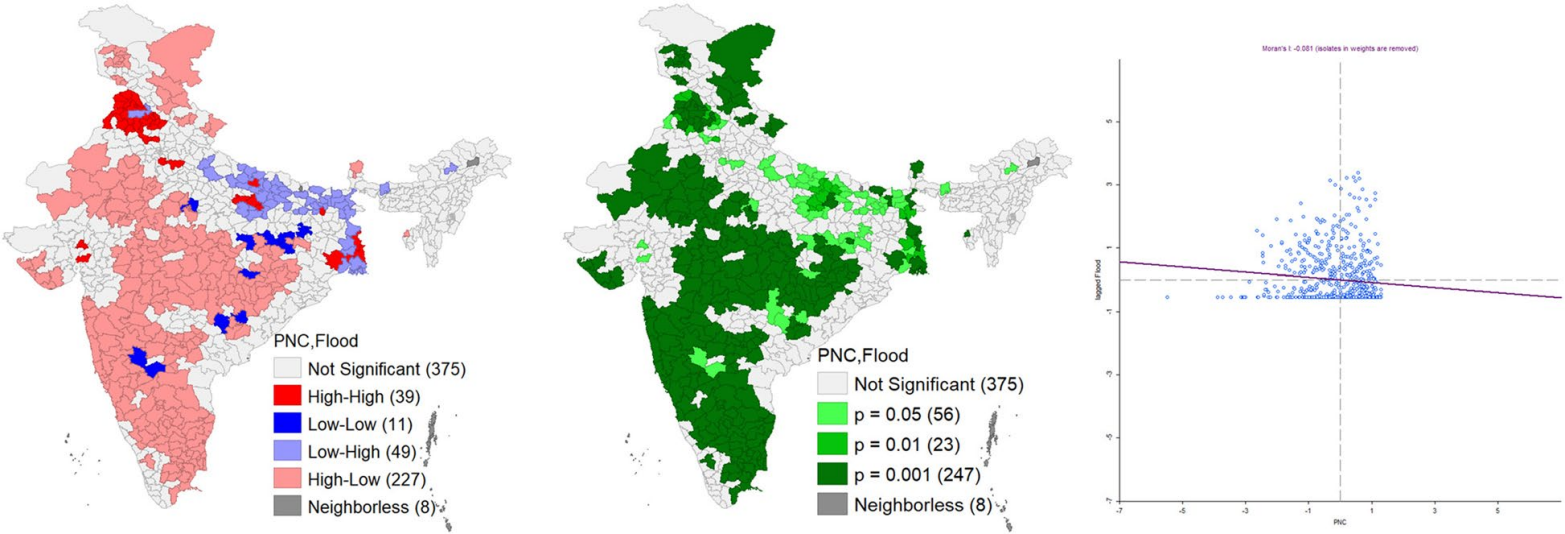
Bivariate Lisa cluster map, significance map and Moran’s-I scatter plot of PNC and flood in India. *Source*: Author’s computation from NFHS 5 data

Figure 5 shows the relationship between flood and PNC access in India. High-high can be observed in Punjab (39 districts), and low PNC and high flood clusters are concentrated in eastern Uttar Pradesh, Bihar and West Bengal (49 districts). While high PNC and low flood clusters were located in the southern and central parts of the country (227 districts). The spatial autocorrelations between flood and PNC were significant in many states like Punjab, Maharashtra, Tamil Nadu, Bihar and West Bengal (Fig. 5). However, it was not significant in Kerala, Assam and Uttar Pradesh. Moreover, negative spatial autocorrelation can be observed from Moran’s Scatter plot. It also depicted that flood-affected districts had lower PNC compare to the non-flooded districts.

**Fig. 6:**
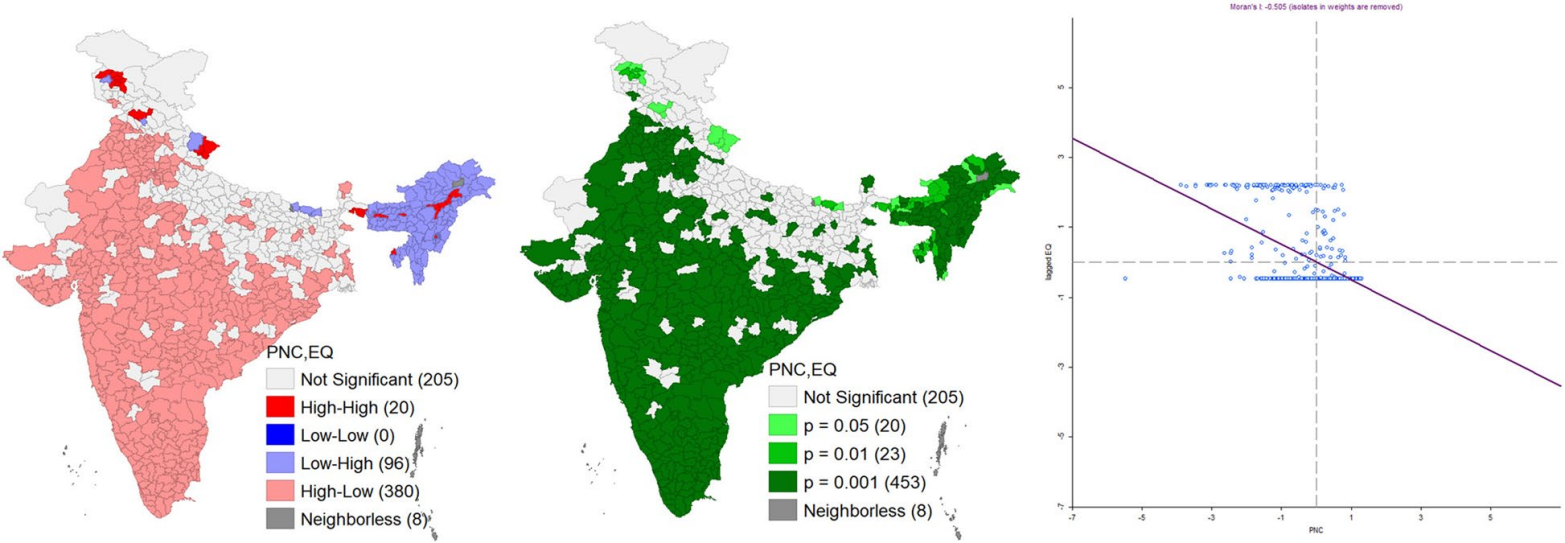
Bivariate Lisa cluster map, significance map and Moran’s-I scatter plot of PNC and earthquake in India. *Source*: Author’s computation from NFHS 5 data

The Moran’s I scatter plot found a strong negative spatial autocorrelation between a severe earthquake and the PNC for neonates (Fig. 6). It shows that most of the northeastern part registered a lower level of PNC along with higher exposure to severe earthquake. While other parts of the country registered a higher PNC having a lower exposure to severe earthquake. Lisa significance map also depicted a significant association between severe earthquake and the PNC among 476 districts (*p* value 0.01).

**Fig. 7:**
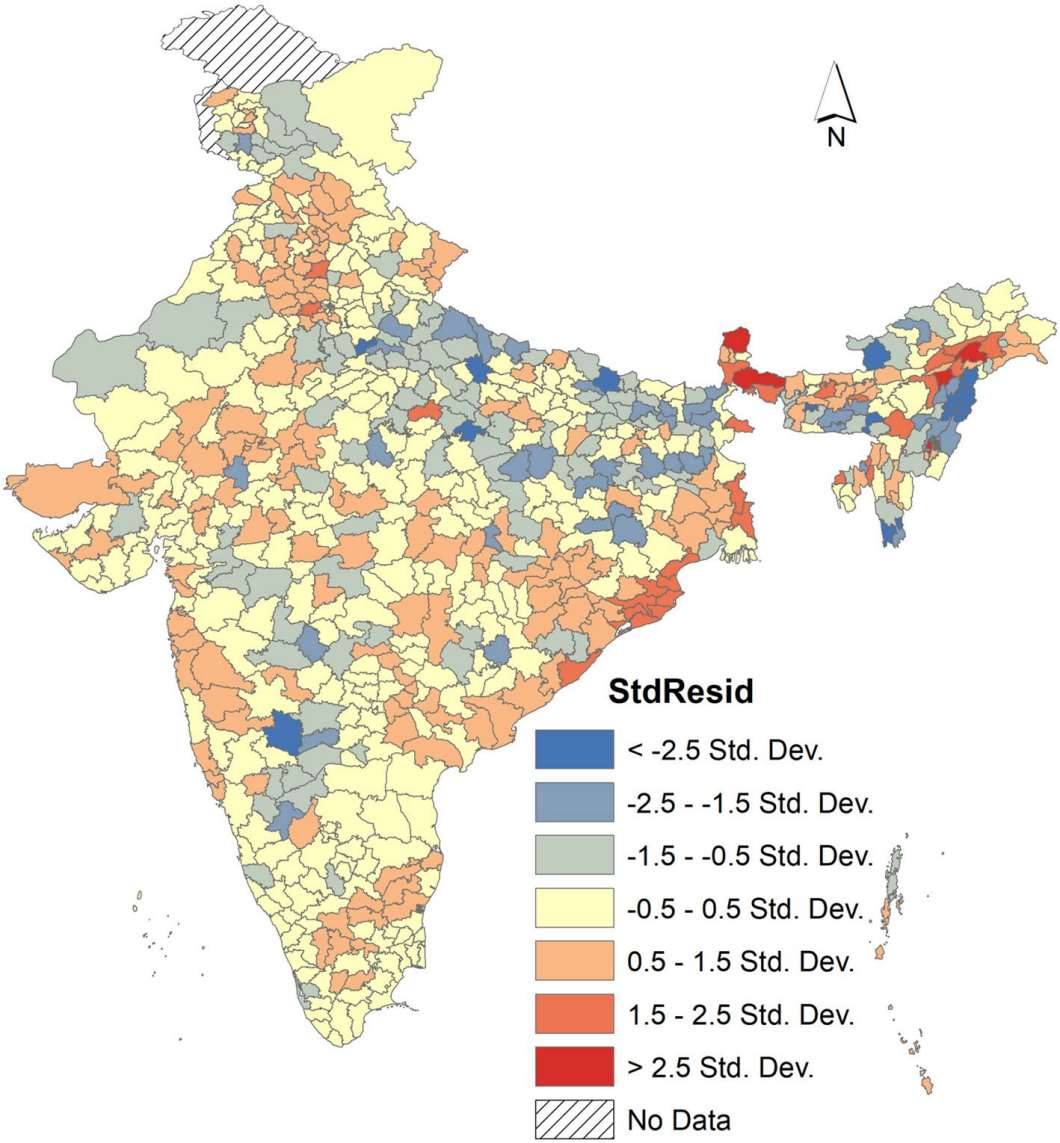
Geographically weighted regression with PNC and other indicators. *Source*: Author’s computation from NFHS 5 data

Geographically Weighted regression also shows the spatially varying relationships between PNC and exposure to hazardous locations such as floods, landslides, and earthquakes (Fig. 7). Here the map represents the underprediction and overprediction of PNC by the exposure to hazards. In 303 districts, the predicted value was very close to the actual value (Std. residual − 0.05 to + 0.05). Some districts in Uttar Pradesh, Bihar, Karnataka, and Nagaland, where PNC is underpredicted (53 districts). On the other hand, districts in Sikkim, West Bengal Odisha, and Haryana it was overpredicted (34 districts). The r square also value depicts that PNC is 60% explained by these three natural hazards.

## Discussion

The association between natural hazards and access to postnatal care is a major public health concern in India, particularly in regions prone to the consequences of natural calamities. However, this area remains unexplored, as no study has specifically addressed this issue in India. Therefore, this study intends to utilize a geospatial approach to investigate the relationship between natural hazards and postnatal care among neonates in India, with the aim of enhancing access to postnatal care and achieving comprehensive coverage.

This study has found that full coverage of postnatal care (PNC) in India is yet to achieve. However, PNC services in hazard-prone areas are much lower than the national average. The severity is much higher in floodplain areas and high-altitude earthquake-prone areas. These findings align with other existing studies [44, 45] and the results of this study also established a strong negative association between postnatal care and natural hazard. Baten et al. [46] demonstrated the impact of incidental and recurrent of any hazards on maternal and postnatal care for neonates. They pointed out that the occurrence of any hazards has a negative impact on maternal healthcare utilization and postnatal care of neonates [46]. However, a significant variation of PNC coverage was found across the regions. It is also evident that areas with multiple hazards are in critical condition in terms of PNC services. Maps and figures have clearly demonstrated that certain regions, i.e., north, east, and northeast, have a higher vulnerability to multiple hazards and lower coverage of postnatal care (PNC). These findings align with previous research examining the association between diverse hazards and health, which likewise yielded unfavourable outcomes [47–51]. Moreover, Portner et al. [52] observed that climate change and natural hazards could increase challenges in managing health issues and providing healthcare services. Mboera [53] asserted that climate change could have immediate and long-term effects on human health and health system therefore preparedness for adverse conditions is very much needed [34].

The most plausible explanation in this context is that in areas prone to natural hazards, it is difficult for women to receive postnatal care due to the damage of healthcare facilities and the interruption in accessing healthcare services [54]. Furthermore, there are several other reasons why postnatal care may be poor in areas affected by natural hazards, first it can disrupt transportation systems, making it difficult to travel to healthcare facilities to receive postnatal care [55]. Second, in areas affected by natural hazards, resources may be diverted to emergency response efforts, leaving fewer resources available for postnatal care services [56]. Third, it can displace populations, making it difficult for women to access healthcare services, including postnatal care [57] as well as, women may be afraid to seek postnatal care services due to concerns about their safety in areas that have recently experienced a natural disaster. Addressing these challenges requires a coordinated effort to rebuild healthcare infrastructure, improve transportation systems, and increase community awareness and education about the importance of postnatal care. Earlier studies have highlighted some of the impact of natural hazards on healthcare accessibility, with some research indicating severe disruption of healthcare services in hazards-prone regions [58–61]. Ahmed & Eklund [62] also noted that natural hazards have negatively impacted healthcare accessibility, resulting in significant challenges for local populations in accessing healthcare services.

Natural hazards and its impact on human health and mortality have been rising as a global concern [63]. Intergovernmental Panel on Climate Change (IPCC) has also periodically reported that the impact of natural hazards on the individual’s livelihood and health [64]. However, these unwelcome natural hazards and their long-term effects sometimes prevent people from accessing basic healthcare facilities. Timely diagnosis, healthcare, or treatment check-ups could prevent child mortality [53]. On the other hand, post-natal care for neonate is very crucial to halt the infant mortality which is still high in India (35 per 1000 live birth). To reduce infant mortality, India steps several programs and policies that are mostly linked to post-natal check of neonates. Some of the important programs are Reproductive and Child Health (RCH) programme in 1997, National Health Mission in 2005, Millennium Development Goal with target completion by 2015 and Sustainable Development Goal with target completion by 2030. Though the Indian government implemented series of interventions towards the reduction of child mortality shows the decline in child or infant mortality, but it is still high at national and some regional level, particularly in the northeastern and eastern regions. Regional disparity in terms of PNC and child mortality is observed in India [65]. It is arguable that despite of series of intervention, several parts of the country still lack in accessing PNC services. Therefore, while studying healthcare utilization it is necessary to consider both socio-economic and geographic obstacles among the population.

Since based on our knowledge none of previous studies had focused on the link between PNC coverage and natural hazard in India and other countries, therefore, it was difficult to raise the findings of previous works, and this was the strength of the current study. However, the current study had some limitations, and thus, it is necessary to exercise caution while interpreting the findings. The cluster points did not provide an accurate location of the PSU or CEB. Therefore, although the point was categorized as hazardous or non-hazardous, some minor errors may have occurred. Nevertheless, we assumed that the point would be in close proximity, resulting in a similar effect. Additionally, the study used cross-sectional data, and the survey did not inquire about the natural hazard and its impact. As a result, this study does not assert that hazards were the definite cause, but rather that an association between PNC coverage and natural hazards existed therefore, generalizability from the result could not be possible; however, using the large sample size we examine the association in detail. Considering the validity of the information on outcome variable, we were unable to externally validate; however, a trends were found in various rounds of the NFHS surveys which ensures the consistency. Owing to the cross-sectional data, a causal inference was failed and a cohort stud would be appropriate in validating our results.

## Conclusion

The present study highlights the substantial geographical variation of PNC coverage between hazardous and non-hazardous areas in India. PNC coverage is one of the crucial problems in the state or district, especially north, east, and northeastern districts prone to multiple natural hazards like flood, landslide and earthquake. Therefore, to achieve the Sustainable Development Goal 3 and reduce the child mortality, region or state specific health program is needed, so that equality in PNC coverage can be promoted. The current study highlights the foremost need for interventions on the dealing with the long-term impact of the natural hazards and immediate recovering the lost existing health facility, so that people may get PNC coverage. Further, intervention is also needed in the development of existing medical health facilities and number of medical staff so that a big chunk of people may get service at one time without delay.

## Data Availability

The data can be obtained using the following links https://dhsprogram.com/data/dataset/India_Standard-DHS_2020.cfm?flag=0 and https://vai.bmtpc.org/.
